# Utrophin influences mitochondrial pathology and oxidative stress in dystrophic muscle

**DOI:** 10.1186/s13395-017-0139-5

**Published:** 2017-10-24

**Authors:** Tahnee L. Kennedy, Lee Moir, Sarah Hemming, Ben Edwards, Sarah Squire, Kay Davies, Simon Guiraud

**Affiliations:** Oxford Neuromuscular Centre, Department of Physiology, Anatomy and Genetics, University of Oxford, Oxford, OX1 3PT UK

**Keywords:** Duchenne muscular dystrophy, Mitochondria defects, Oxidative stress, Utrophin

## Abstract

**Background:**

Duchenne muscular dystrophy (DMD) is a lethal X-linked muscle wasting disorder caused by the absence of dystrophin, a large cytoskeletal muscle protein. Increasing the levels of the dystrophin-related-protein utrophin is a highly promising therapy for DMD and has been shown to improve pathology in dystrophin-deficient mice. One contributing factor to muscle wasting in DMD is mitochondrial pathology that contributes to oxidative stress and propagates muscle damage. The purpose of this study was to assess whether utrophin could attenuate mitochondria pathology and oxidative stress.

**Methods:**

Skeletal muscles from wildtype C57BL/10, dystrophin-deficient *mdx*, dystrophin/utrophin double knockout (*dko)* and dystrophin-deficient *mdx*/utrophin over-expressing *mdx*-Fiona transgenic mice were assessed for markers of mitochondrial damage.

**Results:**

Using transmission electron microscopy, we show that high levels of utrophin ameliorate the aberrant structure and localisation of mitochondria in *mdx* mice whereas absence of utrophin worsened these features in *dko* mice. Elevated utrophin also reverts markers of protein oxidation and oxidative stress, elevated in *mdx* and *dko* mice, to wildtype levels. These changes were observed independently of a shift in oxidative phenotype.

**Conclusion:**

These findings show that utrophin levels influence mitochondrial pathology and oxidative stress. While utrophin deficiency worsens the pathology, utrophin over-expression in dystrophic muscle benefits mitochondria and attenuates the downstream pathology associated with aberrant mitochondrial function.

**Electronic supplementary material:**

The online version of this article (10.1186/s13395-017-0139-5) contains supplementary material, which is available to authorized users.

## Background

Duchenne muscular dystrophy (DMD) is a severe and progressive muscle wasting disorder [1, 2] affecting 1:5000 boys [3]. DMD is first evident early in childhood when boys exhibit ambulatory and balance difficulties. Muscle degeneration leads to loss of ambulation at 8–12 years, and patients succumb to respiratory and/or cardiac insufficiencies in their second or third decade of life [4]. The primary cause of pathology is the lack of the protein dystrophin, a large structural protein located at the sarcolemma, connecting the internal cytoskeleton to the surrounding extracellular matrix that confers membrane stability [5]. In its absence, the sarcolemma is susceptible to contraction-induced injury, propagating numerous secondary pathologies. These include extensive loss of calcium (Ca^2+^) homeostasis, calpain activation, oxidative stress and myofibre degeneration [6–11]. One contributing factor to pathology is aberrant mitochondrial function which drives, in part, oxidative stress and propagates muscle damage [12].

Mitochondria are cytoplasmic double membrane-bound organelles involved in energy conversion, generation of reactive oxygen species (ROS) and Ca^2+^ handling [13, 14]. Involvement of mitochondria in the dystrophic pathology has been well characterised with reduced mitochondrial respiration found in skeletal muscles from dystrophin-deficient (*mdx*) mice [15, 16] and DMD [17] patients. Percival and colleagues also observed a reduced density of sub-sarcolemmal mitochondria and aberrant localisation of inter-myofibrillar mitochondria in *mdx* mice [16]. In vitro, dystrophin-deficient myotubes have been shown to be more susceptible to free radical-induced injury compared to wildtype myotubes [10] and in vivo studies showed elevated lipid peroxidation and antioxidant gene expression [11]. These studies highlight the involvement of oxidative stress in the pathophysiology of *mdx* mice, thought to be due to Ca^2+^-induced mitochondrial dysfunction. In support of this, drug-mediated desensitisation of mitochondria to Ca^2+^ overload improves aspects of dystrophic pathology in *mdx* mice [18]. Studies by Vila and colleagues also implicate mitochondria in membrane repair as they accumulate at the site of rupture; a process that is diminished in *mdx* myofibres (which demonstrate impaired sarcolemmal repair) [19]. These studies highlight that loss of dystrophin leads to mitochondrial dysfunction which in turn plays a significant role in dystrophic pathogenesis.

Utrophin is a structural and functional autosomal parologue of dystrophin [20, 21] that is localised to the sarcolemma during foetal development and confined to the neuromuscular junction (NMJ) in mature muscle [22]. Pre-clinical studies have shown that both transgenic utrophin over-expression and pharmacological modulation using small compounds can prevent pathology in *mdx* mice [23–27]. Thus, modulation of utrophin is a highly promising therapy for DMD patients, currently under investigation in a phase II clinical trial [28]. Given the ability of utrophin to act as a surrogate to compensate for dystrophin deficiency at the sarcolemma and the contribution of aberrant mitochondrial function to the dystrophic pathology, the purpose of this study was to test whether utrophin over-expression or absence could influence mitochondrial pathology caused by dystrophin deficiency. As oxidative stress propagates the dystrophic phenotype and is a downstream effect of mitochondrial dysfunction, markers of oxidative stress were also assessed.

The capacity of utrophin to improve the dystrophic pathology has also been implicated independently from membrane stabilisation. Upregulation of a key metabolic regulator proliferator-activated receptor gamma coactivator (PGC)-1α has been shown to improve *mdx* pathology, likely through stimulation of a slower oxidative phenotype [29, 30]. These improvements were observed alongside elevated utrophin and Sirtuin (Sirt) 1 protein expression levels. Therefore, we have also assessed PCG-1α signalling to determine whether over-expression of utrophin can impact the dystrophic pathology outside of its recognised role as a dystrophin surrogate.

Our overall aim was to better understand the molecular impact of utrophin upregulation and deficiency on dystrophic muscle to inform the optimal clinical application of utrophin-modulation strategies.

## Methods

### Mice

Wildtype C57BL/10ScSnOlaHsd (C57BL/10), dystrophin-deficient C57BL/1010ScSn-*Dmdmdx*/J (*mdx*), dystrophin/utrophin double knockout (*dko*) and dystrophin-deficient/utrophin over-expressing C57/Bl10ScSn-*Dmdmdx*/J-Tg (ACTA1-Utrn)2Ked (*mdx*-Fiona) mice were assessed. C57BL/10 mice were obtained from Envigo (UK) and all other mouse strains were bred in the Biomedical Services facility, University of Oxford. Ten-week-old male mice were sacrificed and muscles were immediately excised and snap frozen (with the exception of those taken for transmission electron microscopy assessment) in liquid nitrogen or embedded in OCT and frozen in thawing isopentane. Samples were stored at − 80 **°**C until further analysis.

### Histological assessment

Serial sections (10 μm) were cut transversely through the extensor digitorum longos (EDL) using a refrigerated (− 20 °C) cryostat (Bright OTF5000 Cryostat, Casa Alvarez Scientific Material, Spain). To assess muscle architecture fresh frozen sections were reacted with haematoxylin and eosin (H&E). Cytochrome oxidase (COX) staining was performed by incubating sections for 1 h at 37 °C in a phosphate buffer (pH 7.6) containing 50 mM sodium dibasic phosphate heptahydrate, 50 mM sodium monobasic phosphate monohydrate, 2.2 M sucrose, 1.3 mM 3,3′-diaminobenzidine tetrahydrochloride and 80 mM cytochrome C. Sites of COX activity were coloured brown [31, 32]. Succinate dehydrogenase (SDH) staining was performed by incubating sections for 15 min in SDH solution containing 50 mM disodium succinate, 0.7 M sodium azide, 6.5 mM disodium EDTA, 13.5 mM NaH_2_PO_4_.H_2_O, 6.8 mM Na_2_HPO_4_.7H_2_O, 1.5 mM nitroblue tetrazolium and 1.1 mM 1-methoxyphenzine methosulfate. Sites of SDH activity were coloured blue [33]. Gomori’s trichome staining was performed by incubating slides for 5 min in 1% glacial acetic acid, 12.8 mM chromotrope 2R, 3.7 mM fast green FCF and 2.1 mM phosphotungstic acid prior to dehydration in ascending concentrations of ethanol. Mitochondria were coloured red. All sections were air dried before application of coverslip and mounting medium and acquisition of digital images (Axioplan 2 Microscope System; Carl Zeiss, Germany).

### Western blot analysis

Muscle samples were homogenised (Polytron 2100; Lucerne, Switzerland) for 3–15 s on ice in RIPA buffer (Sigma-Aldrich) supplemented with protease inhibitors (1:100; Sigma-Aldrich) and sodium orthovanadate (1 mM; Sigma-Aldrich). Following normalisation of protein concentration, homogenates were resolved in Laemmli buffer before heating to 95 °C (with the exception of those assessed using MitoProfile which were heated to 37 °C) for 5 min and were separated by sodium dodecyl sulphate-polyacrylamide gel electrophoresis (SDS-PAGE). For larger molecular weight proteins, utrophin and dystrophin, samples were loaded onto NuPAGE 3–8% TRIS Acetate Midi Gels (Novex, Life Technologies) and transferred overnight to PVDF membranes (Millipore). Membranes were blocked for 1 h with 5% skimmed milk in 0.1% PBS-Tween20 (T) and then incubated with primary antibodies in 0.1% PBS-T for 1 h at room temperature. Primary antibody and dilutions used were: utrophin, (MANCHO3; 84A) 1:50, a gift from G.E. Morris and dystrophin (Abcam; ab15277) 1:50. For all other targets, samples were loaded onto Criterion TGX (Tris-Glycine eXtended) pre-cast gels (Bio-Rad Laboratories, CA, USA) and were transferred for 4 h to PVDF membranes (Millipore). Membranes were blocked for 1 h with 5% skimmed milk in 0.1% PBS-T and then incubated with primary antibodies in 0.1% PBS-T overnight at 4 °C. Primary antibodies and dilutions used were Sirt1 (Millipore; 07–131) 1:500, AMPK (Cell Signaling Technologies; 9272S) 1:1000, phosphorylated AMPK (Cell Signaling Technologies; 9271S) 1:1000 and MitoProfile® Total OXPHOS Rodent WB Antibody Cocktail (Abcam; 110,413) 1:1000. For all targets, antibody binding was detected with horseradish peroxidase (HRP)-conjugated immunoglobulin and visualised by chemiluminescent detection (ECL Prime Western Blotting detection reagent Amersham) and imaging system ImageQuant LAS 4000 (GE Healthcare Life Sciences). Band densities were quantified with the Fiji ImageJ 1.49i software and normalised to total protein content of samples. Total protein was assessed by incubating membrane in Ponceau S solution (Sigma-Aldrich) followed by imaging using the ImageQuant LAS 4000 (GE Healthcare Life Sciences). Lane densities were quantified with the Fiji ImageJ 1.49i software. Total protein staining was used as the loading control as expression of housekeeping genes are not stable across genotypes.

### Transmission electron microscopy (TEM)

Ultrastructural analysis was performed on tibialis anterior (TA) muscle samples fixed in 2.5% glutaraldehyde and 2–4% paraformaldehyde in 0.1 mM cacodylate buffer (pH 7.4) at room temperature for 1–2 h and then at 4 °C for 24 h. Samples were washed with 0.1 mM sodium cacodylate buffer 4 × 15 min, with the third wash also containing 25 mM glycine. Samples were post-fixed with 2% osmium, 1.5% potassium ferrocyanide in 0.1 M sodium cacodylate buffer for 1 h with rotation at 4 °C. Samples were washed 4 × 15 min with water, en bloc stained with 0.5% uranyl acetate overnight at 4 °C, washed with water and dehydrated 3–4 h using a graded ethanol series. Samples were infiltrated with TAAB TLV epoxy resin according to the following schedule: 1:3 resin:ethanol for 2 h, 1:1 resin:ethanol for 3 h, 3:1 resin:ethanol for 1 h, overnight in 100% resin, followed by 6 more changes of 100% resin over 36 h before embedding in beem capsules and polyermising for 48 h at 60 °C. Ultrathin sections (60 nm) were post-stained for 5 min with lead citrate, washed with water and examined using a FEI Tecnai 12 transmission electron microscope (OR, USA) operated at 120 kV with a Gatan OneView CMOS camera (CA, USA).

### Immunofluorescence

Fresh frozen muscle cross-sections (10 μm thick) assessed of utrophin and dystrophin were blocked for 30 min with 10% foetal bovine serum (FBS)/PBS and then incubated with primary antibodies in 5% FBS/PBS overnight at 4 °C. Primary antibodies and dilutions used were utrophin (developed in-house as previously described [34]) 1:2000 and dystrophin (Abcam; ab15277) 1:2000. Sections were reacted with Alexa Fluor ® 488 donkey anti-goat IgG antibody (ThermoFisher Scientific; A-11055) or Alexa Fluor ® 594 donkey anti-rabbit IgG antibody (ThermoFisher Scientific; R37117) 1:2000 for 1 h at room temperature and then rinsed in PBS before air drying and application of cover slip with fluorescent mounting medium. Intensity was detected using a fluorescence microscope (Axioplan 2 Microscope System; Carl Zeiss, Germany).

### Immunoglobulin (Ig) G staining for assessment of membrane integrity

Fresh frozen muscle cross-sections were blocked for 30 min with 10% FBS/PBS and then incubated with Alexa Fluor ® 488 goat anti-mouse IgG (Life Technologies; ab150117) antibody, 1:750 in 5% FBS/PBS overnight at 4 °C. Sections were rinsed in PBS before air drying and application of cover with fluorescent mounting medium, intensity was detected using a fluorescence microscope (Axioplan 2 Microscope System; Carl Zeiss, Germany).

### Gene expression analysis

PGC-1α and PGC-1β mRNA expression were determined by quantitative real-time polymerase chain reaction (qPCR) as previously described [27]. Total RNA was extracted from 10 to 20 mg of TA muscle using TRIzol reagent as per manufacturer’s recommendations. Two hundred fifty nanogram RNA was transcribed into cDNA using the QuantiTect Reverse Transcription kit (Qiagen 205313), and the resulting cDNA was stored at − 20 °C for subsequent analysis. qPCR was performed using the StepOnePlus™ Real-Time PCR system (Applied Biosystems) with SYBR® Fast Master Mix (Thermofisher 4385612). Primer sequences used were PGC-1α (FWD, 5′-AAGTGTGGAACTCTCTGGAACTG-3′; REV, 5′-GGGTTATCTTGGTTGGCTTTATG-3′; PGC-1β (FWD, 5′-TGCTGCTGTCCTCAAATACG-3′, REV, 5′-TGGAGACTGCTCTGGAAGGT-3′). Gene expression was normalised to ribosomal protein S13 (FWD; 5′-CCCCGAGGATCTCTACCATT-3′, REV; 5′-GCCACTAGACAGAGGCTGT-3′). Results were analysed using the ΔΔCT method.

### Spectrophotometric enzyme assays

Quadriceps muscles were crushed in liquid nitrogen and suspended in homogenisation buffer (pH 7.4) containing 100 mM KCl, 50 mM MOPS and 0.5 mM EGTA. For assessment of complex I activity, 2.5 mg of tissue was added to 1 mL assay buffer heated to 30 °C containing 0.25 M potassium phosphate buffer (pH 7.4; potassium phosphate dibasic and potassium phosphate monobasic), 5 mM MgCl_2_, 0.13 mM NADH, 65 μM Coenzyme Q_1_, 2.5 mg fatty acid-free bovine serum albumin per reaction and 3.6 mM Antimycin A. Change in absorbance was read at 340 nm. For assessment of complex II activity, 2.5 mg of protein was added to 0.25 M potassium phosphate buffer (pH 7.4; potassium phosphate dibasic and potassium phosphate monobasic), 5 mM MgCl_2_ and 2 mM sodium succinate dibasic hexahydrate followed by a 10 min incubation at 30 °C prior to the addition of 2 μg/mL Antimycin A, 2 μg/mL rotenone and 50 uM dichlorophenolindoprol (DCIP). Change in absorbance was read at 600 nm. For both assays, absorbance was read using a Pharmaspec UV-1700 UV visible spectrophotometer (Shimadzu, Kyoto Japan) and UV 2.10 software (Shimadzu). Activity of complex I and II was determined by the gradient of change in absorbance relative to the extinction coefficient of NADH and DCIP, respectively. Assays were validated by the addition of specific complex inhibitors, rotenone (complex I) and oxaloacetate (complex II). All reagents used for these assays were obtained from Sigma.

### Oxidation indicator dihydroethidium (DHE) intensity

Fresh frozen muscle cross-sections (10 μm thick) were incubated in 2 μM DHE (Cayman Chemical, MI, USA) in PBS with 0.1% DMSO at 37 °C for 30 mins. Sections were rinsed in PBS before air drying and application of cover with fluorescent mounting medium containing DAPI. DHE intensity was detected as red fluorescence using a fluorescence microscope (Axioplan 2 Microscope System; Carl Zeiss, Germany).

### Protein oxidation detection

OxyBlot™ Protein Oxidation Detection Kit (Millipore S7150) was utilised to perform immunoblot detection of carbonyl groups introduced into proteins by oxidative reactions. Samples were prepared and assessed as per manufacturer’s instructions. Briefly, fresh frozen muscle tissue was homogenised, as described previously, and protein concentrations normalised. Carbonyl groups in protein side chains were derivatised by reaction with 2, 4-dinitrophenylhydrazine (DNPH) and samples separated using SDS-PAGE. For separation, samples were loaded onto Criterion TGX (Tris-Glycine eXtended) pre-cast gels (Bio-Rad Laboratories, CA, USA) and were transferred for 4 h to PVDF membranes (Millipore). Membranes were blocked for 1 h with 5% skimmed milk in 0.1% PBS-T and then incubated with primary antibody (provided) in 0.1% PBS-T overnight at 4 °C. Antibody binding was detected with HRP-conjugated immunoglobulin and visualised by chemiluminescent detection (ECL Prime Western Blotting detection reagent Amersham) and imaging system ImageQuant LAS 4000 (GE Healthcare Life Sciences). Band densities were quantified with the Fiji ImageJ 1.49i software and normalised to total protein content of samples. Lanes with higher intensity band densities indicate a greater degree of protein carbonylation (a type of oxidation that can be promoted by ROS) [35].

### Statistical analysis

Results were analysed using Prism (GraphPad Software, Inc.). One-way analysis of variance (ANOVA) was used to compare groups and Tukey’s post hoc test was used to determine significant differences between individual groups. The level of significance was set at *P* < 0.05 for all comparisons. All values are presented as mean ± S.E.M.

## Results

### Loss of dystrophin and utrophin from the sarcolemma leads to membrane rupture

**Fig. 1:**
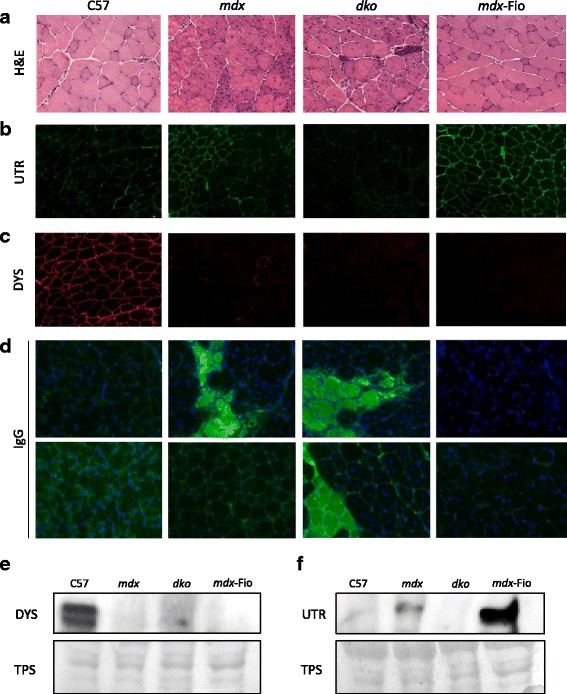
Muscle architecture and dystrophin and utrophin expression in extensor digitorum longus (EDL) muscles from C57BL/10, *mdx*, *dko* and *mdx*-Fiona mice. Representative images of (**a**) muscle architecture (assessed by H&E staining), (**b**) utrophin (UTR) immunofluorescence, (**c**) dystrophin (DYS) immunofluorescence and (**d**) membrane integrity (assessed via IgG infiltration and indicated by green) of EDL muscle cross-sections from C57BL/10 (C57), *mdx*, *dko* and *mdx*-Fiona (*mdx*-Fio) mice (*n* = 5–6/group). Protein expression levels, detected by western blot, of (**e**) dystrophin and (**f**) utrophin in quadriceps muscles of C57, *mdx*, *dko* and *mdx*-Fio mice and corresponding loading control (total protein stain; TPS; each lane contains 3 pooled samples from each genotype)

Prior to the investigation of mitochondrial pathology, we first confirmed the phenotypes of the mouse strains used. Assessment of muscle architecture via H&E staining of EDL muscles revealed the expected phenotypes, with dystrophic legions evident in *mdx* muscle, worsened in *dko* muscle and ameliorated in *mdx*-Fiona muscle (Fig. 1a). Utrophin immunofluorescence (IF) revealed the presence of utrophin at the NMJ in C57BL/10 muscle, the NMJ and in regenerating fibres in *mdx* muscle and uniformly localised to the sarcolemma of *mdx*-Fiona muscle. As expected, utrophin was absent from *dko* muscle (Fig. 1b). Dystrophin was present only in C57BL/10 mice and absent from all other strains (Fig. 1c). IgG infiltration was observed in some *mdx* mice, was evident in all *dko* mice and not present in C57BL/10 and *mdx*-Fiona animals (Fig. 1d). Western blot assessment revealed the expected results. Dystrophin was present only in C57BL/10 mice. Utrophin levels were elevated in *mdx*-Fiona mice compared to *mdx* mice, detected in low levels in C57BL/10 mice and absent from *dko* mice (Fig. 1e–f).

### Aberrant mitochondrial structure and localisation are evident in *mdx* and *dko* mice and rescued in *mdx*-Fiona mice

**Fig. 2:**
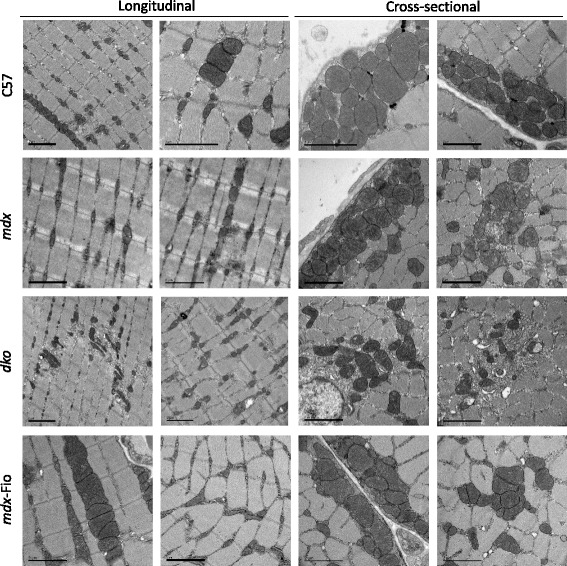
Transmission electron micrographs of tibialis anterior muscles from C57BL/10, *mdx*, *dko* and *mdx*-Fiona mice. Representative images of longitudinal sections and cross-sections of tibialis anterior muscles from C57BL/10 (C57), *mdx*, *dko* and *mdx*-Fiona (*mdx*-Fio) mice (*n* = 3/group). All scale bars = 2 μm, magnification varies

**Fig. 3:**
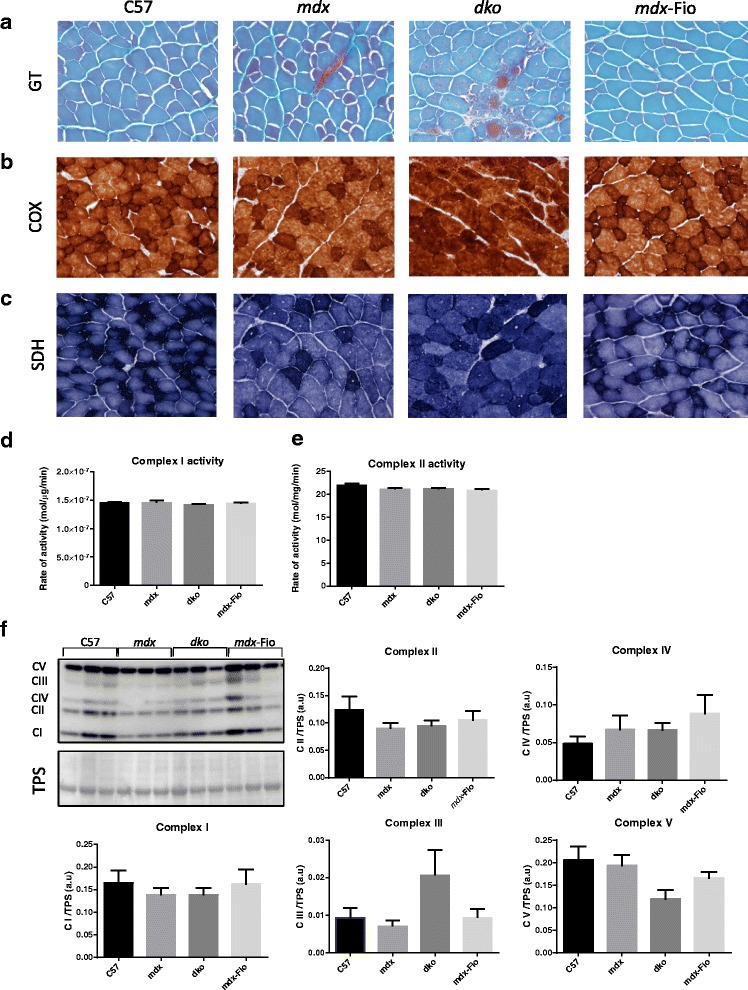
Assessment of markers of oxidative capacity in C57BL/10, *mdx*, *dko* and *mdx*-Fiona mice. Representative images of extensor digitorum longos muscles from C57BL/10 (C57), *mdx*, *dko* and *mdx*-Fiona (*mdx*-Fio) mice assessed for (**a**) mitochondrial aggregation (Via Gomori’s trichrome; GT), (**b**) cytochrome oxidase (COX) activity and (**c**) succinate dehydrogenase (SDH) activity indicated by increased intensity of red, brown and blue staining, respectively (*n* = 5–6/group). Enzyme activity of mitochondria respiratory chain complexes (**d**) I and (**e**) II (*n* = 4/group). **f** Representative images and quantification of protein expression in mitochondria respiratory chain complexes I–V in quadriceps muscles from C57, *mdx*, *dko* and *mdx*-Fio mice, normalised to loading control (total protein stain (TPS)). Data are represented as mean ± SEM, no differences were observed between groups. Representative image *n* = 3/group, quantification generated from *n* = 9/group

In order to assess mitochondrial sub-structure and localisation, TA muscles from each mouse strain were investigated using TEM. Longitudinal sections of TA muscles from C57BL/10 mice exhibited localisation of mitochondria to the I band of myofibres and sound cristae formation within the mitochondria. Healthy structure was also observed in cross-sectional images (Fig. 2 and Additional file 1: Figure S1). Mitochondria were present at the I band in *mdx* muscle; however they also displayed abnormal aggregation, localisation to areas of necrosis and compromised structure (Fig. 2, Additional file 2: Figure S2). The aberrant features present in *mdx* mice were exacerbated in *dko* mice. Clear mitochondrial delocalisation, aggregation in regions of necrosis and severe disruption of internal membrane structure were all evident (Fig. 2, Additional file 3: Figure S3). TEM analysis of *mdx-*Fiona muscle did not reveal markers of pathology, with correct localisation and intact internal mitochondria structure (Fig. 2, Additional file 4: Figure S4). Histological assessment further supported TEM results with Gomori’s trichrome staining revealing increased mitochondrial aggregation in regions of necrosis in the EDL muscles from *mdx* and *dko* mice (Fig. 3a). Muscles from *mdx*-Fiona mice were comparable to C57BL/10 mice (Fig. 3a). These results show that utrophin is able to prevent the damage to mitochondria that results from dystrophin deficiency.

### Mitochondrial benefits are independent from oxidative phenotype in *mdx*-Fiona mice

COX and SDH staining and enzyme activity assessment of mitochondrial respiratory chain complexes I and II were employed to test oxidative capacity of EDL muscles. COX and SDH staining, indicated by brown and blue colouring respectively, were not altered in *mdx*-Fiona mice compared to C57BL/10 animals. Muscle cross-sections from *dko* mice did not exhibit discrete staining of myofibres but rather, for both SDH and COX activity, overall darker staining compared to C57BL/10, *mdx* and *mdx*-Fiona mice (Fig. 3b–c). In support of these findings, specific enzymes activity of respiratory chain complexes I and II (Fig. 3d, e) and protein expression of complexes I–V (Fig. 3f) showed no significant difference between groups. Due to high levels of variation in protein expression, the sample number was increased to 9 for assessment of mitoprofile. However, statistical differences were still not resolved. These findings suggest oxidative capacity does not impact mitochondrial health in this context.

### Mitochondria-associated cell signalling was altered in *mdx* and *dko* mice

**Fig. 4:**
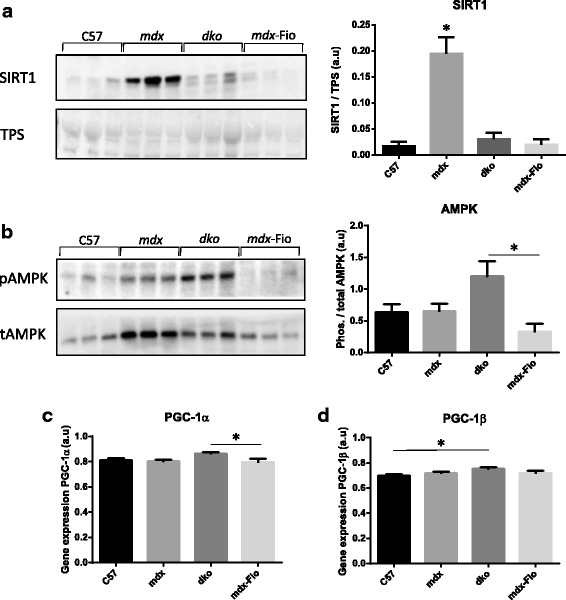
Assessment of mitochondria-associated cell signalling in C57BL/10, *mdx*, *dko* and *mdx*-Fiona mice. Representative images and quantification of **a** Sirtuin 1 (Sirt 1), normalised to loading control (total protein stain; TPS), and **b** phosphorylation status of AMP-activated protein kinase (AMPK), normalised to total protein expression of AMPK, in quadriceps muscles from C57BL/10 (C57), *mdx*, *dko * and *mdx*-Fiona (*mdx*-Fio) mice. Gene expression of **c** proliferator-activated receptor gamma coactivator (PGC) -1α and **d** PGC-1β in tibialis anterior muscles from C57, *mdx*, *dko* and *mdx*-Fio mice. Data are represented as mean ± SEM, * denotes significance set at *P* < 0.05. Representative image *n* = 3/group, quantification generated from *n* = 5-6/group

Assessment of the Sirt1/PGC-1α/AMP-activated protein kinase (AMPK) signalling axis, implicated in metabolic control and mitochondrial biogenesis [36] revealed elevated expression in *mdx* and *dko* mice compared to C57BL/10 and *mdx*-Fiona mice. Western blot analysis of quadriceps muscles revealed significantly elevated Sirt1 protein levels in *mdx* mice compared to C57/BL10, *dko* and *mdx*-Fiona mice (Fig. 4a). AMPK activation was assessed by comparisons between phosphorylated and total protein expression levels. Western blot analysis revealed that total AMPK was elevated in *mdx* and *dko* mice. However, the relative levels of phosphorylation were only significantly different in muscle homogenates from *dko* mice compared to *mdx*-Fiona mice (Fig. 4b).Given the capacity of PGC-1β to compensate for PGC-1α [37], gene expression analysis of both was next assessed. PGC-1α was slightly elevated in *dko* mice compared to *mdx*-Fiona mice. No significant differences were observed between remaining genotypes (Fig. 4c). Assessment of PGC-1β revealed slightly elevated levels in *dko* mice compared to C57BL/10 mice, no significant differences were observed between remaining genotypes (Fig. 4c). Components of this signalling axis are elevated in *mdx* and *dko* mice and maintained at wildtype levels in *mdx*-Fiona mice; however this signalling does not appear to be involved in the preservation of mitochondrial health observed in *mdx*-Fiona mice.

### Markers of oxidative stress are elevated in *mdx* and *dko* mice and reduced in *mdx*- Fiona mice

**Fig. 5:**
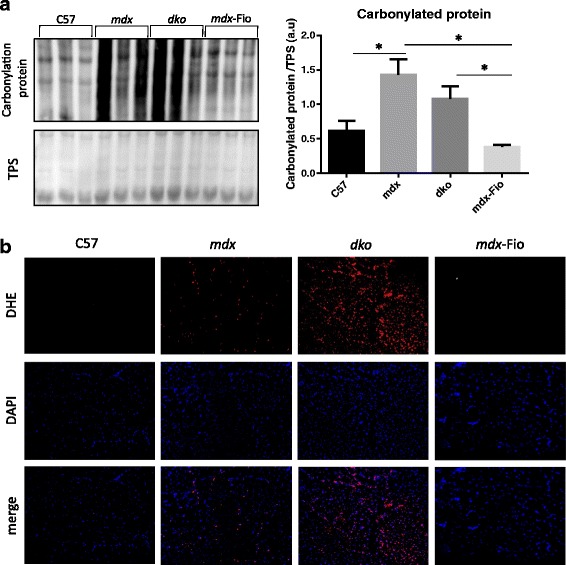
Protein carbonylation and oxidative indicator dihydroethidium (DHE) intensity in C57BL/10, *mdx*, *dko* and *mdx*-Fiona mice. **a** Representative images and quantification of carbonylated proteins (indicated by more intense banding), normalised to loading control (total protein stain (TPS)) in quadriceps muscles from C57BL/10 (C57), *mdx*, *dko* and *mdx*-Fiona (*mdx*-Fio) mice. **b** Representative images of extensor digitorum longos muscle cross-sections reacted with DHE and DAPI from C57, *mdx*, *dko* and *mdx*-Fiona mice. Data are represented as mean ± SEM, * denotes significance set at *P* < 0.05. Representative image *n* = 3/group, quantification generated from *n* = 6/group

Assessment of carbonylated protein content in quadriceps muscle homogenates were assessed using the oxyblot assay. Carbonylated protein levels were elevated in *mdx* mice compared to C57BL/10 mice. *mdx*-Fiona mice presented a very similar profile to wildtype animals. In contrast, *dko* mice exhibited elevated levels of protein carbonylation compared to *mdx*-Fiona mice, but levels were not significantly different to protein carbonylation in C57BL/10 mice. Protein carbonylation levels were not different between *mdx* and *dko* mice (Fig. 5a). These findings were supported by assessment of the oxidation indicator DHE, elevated in EDL cross-sections from *mdx* and *dko* mice compared to C57BL/10 and *mdx*-Fiona mice (Fig. 5b). Overall, markers of oxidative stress were increased in *dko* and *mdx* mice and maintained at wildtype levels in *mdx*-Fiona mice.

## Discussion

The data presented here show that increased utrophin expression ameliorates the aberrant mitochondrial structure and mitochondrial localisation observed in *mdx* and *dko* mice. Elevated utrophin in dystrophic muscle also reverted markers of protein carbonylation and oxidative stress to wildtype levels. These changes were observed independently from a shift in oxidative phenotype. Together, these findings show that utrophin over-expression benefits mitochondria in dystrophic muscle and attenuates the downstream pathology associated with aberrant mitochondrial function. Utrophin is expressed from early development in the transgenic models used here whereas utrophin modulation using viral vectors or small drugs can only be administered from birth [23]. However, these data reinforce the view that utrophin has the potential to compensate for dystrophin deficiency in DMD patients.

Previously, we have shown that both transgenic and pharmacological modulation of utrophin improves the dystrophic pathology [23, 26, 27]. Over-expression of the full-length utrophin ameliorated the dystrophic histopathology and improved membrane integrity. In order to assess the impact of utrophin on the sub-cellular structure of dystrophic muscle, TEM was utilised. Using similar techniques, Percival and colleagues showed that mitochondria in *mdx* mice exhibit aberrant structure, delocalisation from the I band and cluster in regions of necrosis [16]. Our findings emulate these observations, with mitochondria in *mdx* mice frequently localised to regions of necrosis and loss of cristae formation. We also observed that additional loss of utrophin, in *dko* mice, worsened these parameters with more extensive delocalisation and breakdown of internal structure. TEM images of muscles from *mdx*-Fiona mice were comparable to healthy muscles from C57BL/10 mice. These observations were further supported by Gomori’s trichrome staining which revealed aggregation of mitochondria in muscles from *mdx* and *dko* mice but not in those from *mdx*-Fiona and C57BL/10 mice. Our findings support previous studies that demonstrate dystrophin efficiency impairs spatial control of mitochondria and elicits mitochondrial damage [16]. Furthermore, we show for the first time these features are exacerbated by utrophin deficiency. Transgenic over-expression of utrophin was able to attenuate the above parameters, indicating utrophin can effectively replace dystrophin at the sarcolemma preventing mitochondrial damage.

Given the improved mitochondrial structure, it might be expected that the PGC-1α signalling pathway would be elevated in muscles from *mdx*-Fiona mice compared to *mdx* and *dko* mice. However, the opposite was observed, indicating the elevated signalling of this pathway is associated with the dystrophic phenotype and not mitochondrial health. As *mdx* mice undergo prolonged bouts of degeneration and regeneration, elevated Sirt1 signalling may be associated with mitophagy of damaged myofibres and mitochondrial biogenesis of regenerating myofibres. This may also be the case for elevated AMPK activation and PGC-1α/β expression in muscles from *dko* mice, although regeneration may contribute to a lesser extent. Overall, we see altered signalling associated with mitochondria in *mdx* and *dko* mice, and this was restored to wildtype levels with utrophin over-expression in dystrophic muscle. However, the changes observed in the PGC-1α signalling pathway are unlikely to contribute to the improvements observed in mitochondrial pathology.

High levels of utrophin are associated with a slower, more oxidative, muscle fibre type phenotype which is protective of dystrophic muscle [29, 38]. To determine whether a shift in fibre type profile contributed to the improved mitochondrial parameters observed using TEM, markers of oxidative capacity were tested across genotypes. No differences in COX staining, SDH staining, enzyme activity of respiratory chain complexes I and II or mitochondrial respiratory chain complex protein expression were observed in *mdx*-Fiona mice compared to *mdx* mice. However, similar to a previous study, SDH and COX staining of *dko* muscle appeared more intense compared to wildtype muscle [39]. This is likely to be a product of the severe phenotype in which fast twitch fibres are preferentially lost and not due a direct action on mitochondria [40]. Therefore, it does not appear that increasing utrophin expression impacts oxidative capacity of dystrophic muscle. Although we see no difference in COX or SDH staining in *mdx* mice compared to C57BL/10 mice, which is consistent with previous findings [7, 16, 17], transgenic upregulation of PGC-1α is able to elevate the proportion of slow twitch myofibres in *mdx* mice to above that of C57BL/10 mice [29]. Hence, the lack of reduced activity in *mdx* mice should not impede a transition to a slower fibre type profile in *mdx*-Fiona mice.

Although our observations regarding COX and SDH staining were concordant with previous studies, expression patterns of mitochondrial respiratory chain complex proteins were not. Percival and colleagues have previously observed elevated expression of complex III in *mdx* muscle compared to C57BL/10 muscle [16]; however, Ryu and colleagues saw reduced levels of complexes I, II, IV and V in *mdx* muscles compared to C57BL/10 mice [41]. The discrepancies between these studies and our own may be accounted for by the highly dynamic nature of mitochondria [42]. It is possible the turnover of these proteins may mask disease impact upon mitochondria. Therefore, measurements of the mitochondria respiratory chain complexes may not be a reliable indicator of the metabolic state of mitochondria. Taken together, these findings suggest transgenic upregulation of utrophin does not impact oxidative capacity in the dystrophic context. Therefore, oxidative phenotype is not contributing factor to improved mitochondrial pathology observed in *mdx*-Fiona mice.

Oxidative stress is a prominent feature of the dystrophic pathology. *mdx* mice were previously described as more susceptible to ROS-mediated cell damage and exhibited higher levels of lipid peroxidation compared to wildtype mice [10]. Oxidative injury was also found to precede myofibre necrosis in *mdx* mice, indicating it is likely one to be of the initial phases of pathology [11]. Consequently, the development of antioxidants for the treatment of DMD has been a substantial line of research with numerous compounds investigated, e.g. coenzyme Q_10_, catalase, green tea extract, Epigallocatechin gallate, BN82270, Idebenone, melatonin and N-acetylcysteine [14, 43–49]. Mitochondrial Ca^2+^ overload is known to promote ROS generation and membrane permeability in healthy muscle [13, 50]. As mitochondrial dysfunction is prominent in dystrophic muscle, it is likely to be one major mechanism of oxidative stress in DMD. Here, we show oxidative stress and protein carbonylation were elevated in *mdx* and *dko* mice compared to C57BL/10 mice. This effect was prevented in *mdx*-Fiona mice where parameters were comparable with wildtype.

Given utrophin is localised to the sarcolemma and not the cytosol, reductions in oxidative stress are not likely to be due to a scavenging action but rather a downstream effect of improved membrane integrity. Dystrophic myofibres are more susceptible to membrane rupture compared to healthy muscle fibres, allowing for excessive influx of Ca^2+^ rich extracellular fluid into the cytosol [7–9]. Given the link between Ca^2+^ overload and mitochondrial ROS production and prevention of IgG infiltration observed in *mdx-*Fiona mice, this is likely to be the mechanism of the attenuation of oxidative stress. Although IgG infiltration was not present in all *mdx* muscles, histology can only capture that state of the muscle at the time of harvest. It may be that these mice experience sarcolemmal rupture prior to muscle excision and this elicited mitochondrial stress. Although speculative, this seems the most likely mechanism of elevated oxidative stress. Irrespective of mechanism, our findings demonstrate the antioxidant effect of utrophin in dystrophic muscle. DMD is recognised as having multiple secondary pathologies and of these oxidative stress features prominently. A fuller understanding of the capacity of a specific treatment to target different aspects of pathology will inform the strongest approach for combinatorial treatment.

We recently identified numerous serological biomarkers that are elevated in *mdx* mice compared to wildtype mice; many of these are mitochondria-associated proteins [51]. Heat shock protein (HSP) 60, cytochrome C (CYCS), Sirt 2, HtrA serine peptidase 2 (HTRA2) and mitochondrial import inner membrane translocase subunit TIM14 (DNAJC19) were all elevated in serum from *mdx* mice and importantly normalised to wildtype levels in *mdx*-Fiona mice. These findings further support the capacity of high levels of utrophin to impact mitochondria in dystrophic muscle. Taken in conjunction with our current findings, mitochondrial biomarkers are highlighted as a valuable tool for assessing pre-clinical studies and potentially clinical benefits following treatment with small utrophin-modulating drugs.

## Conclusions

Here, we show that utrophin over-expression in dystrophic muscle improves mitochondrial structure/localisation and reduces oxidative stress and that these benefits are potentially due to improved membrane integrity. We also show a detrimental impact on these parameters in *dko* mice, emphasising the importance of utrophin. Our results highlight the effectiveness of utrophin in alleviating mitochondrial pathology resulting from dystrophin deficiency. Additionally, we demonstrate the antioxidant effect of utrophin modulation, further elucidating its molecular impact on dystrophic muscle and relevance in multi-faceted treatment approaches for DMD patients.

## Additional files

Additional file 1: Figure S1. Transmission electron micrographs of tibialis anterior (TA) muscles from C57BL/10 (C57) mice. Cross-sections of TA muscles from C57 mice. Scale bars = (**A**) 10 μm, (**B**) 2 μm and (**C**) 1000 nm. (*n* = 3; C57 1, C57 2, C57 3). (PPTX 787 kb)

Additional file 2: Figure S2. Transmission electron micrographs of tibialis anterior (TA) muscles from *mdx* mice. Cross-sections of TA muscles from *mdx* mice. Scale bars = (**A**) 10 μm, (**B**) 2 μm and (**C**) 1000 nm. (*n* = 3; *mdx* 1, *mdx* 2, *mdx* 3). (PPTX 805 kb)

Additional file 3: Figure S3. Transmission electron micrographs of tibialis anterior (TA) muscles from *dko* mice. Cross-sections of TA muscles from *dko* mice. Scale bars = (**A**) 10 μm, (**B**) 2 μm and (**C**) 1000 nm. (*n* = 3; *dko* 1, *dko* 2, *dko* 3). (PPTX 841 kb)

Additional file 4: Figure S4. Transmission electron micrographs of tibialis anterior (TA) muscles from *mdx*-Fiona (*mdx*-Fio) mice. Cross-sections of TA muscles from *mdx*-Fio mice. Scale bars = (**A**) 10 μm, (**B**) 2 μm and (**C**) 1000 nm. (*n* = 3; Fio 1, Fio 2, Fio 3). (PPTX 771 kb)

## Abbreviations

AMPK: AMP-Activated protein kinase
ANOVA: One-way analysis of variance
C57BL/10: C57BL/10ScSnOlaHsd
Ca^2+^: Calcium
COX: Cytochrome oxidase
CYCS: Cytochrome C
DCIP: Dichlorophenolindoprol
DHE: Dihydroethidium
*dko*: Dystrophin/utrophin double knockout
DMD: Duchenne muscular dystrophy
DNAJC19: Mitochondrial import inner membrane translocase subunit TIM14
DNPH: 2, 4-dinitrophenylhydrazine
EDL: Extensor digitorum longos
FBS: Foetal bovine serum
H&E: Haematoxylin and eosin
HRP: Horseradish peroxidase
HSP: Heat shock protein
HTRA2: HtrA serine peptidase 2
IF: Immunofluorescence
Ig: Immunoglobulin
*mdx*: Dystrophin-deficient C57BL/1010ScSn-*Dmdmdx*/J
*mdx*-Fiona: Dystrophin-deficient/utrophin over-expressing C57/Bl10ScSn-*Dmdmdx*/J-Tg (ACTA1-Utrn)2Ked
NMJ: Neuromuscular junction
PGC: Proliferator-activated receptor gamma coactivator
qPCR: Quantitative real time-polymerase chain reaction
ROS: Reactive oxygen species
SDH: Succinate dehydrogenase
SDS-PAGE: Sodium dodecyl sulphate-polyacrylamide gel electrophoresis
Sirt: Sirtuin
T: Tween20
TA: Tibialis anterior
TEM: Transmission electron microscopy
UK: United Kingdom
