# Supplementary figures and images for: Utrophin influences mitochondrial pathology and oxidative stress in dystrophic muscle
Source: Skelet Muscle. 2017 Oct 24;7:22. doi: 10.1186/s13395-017-0139-5 (PMC5655821; doi:10.1186/s13395-017-0139-5)

## Slide 1
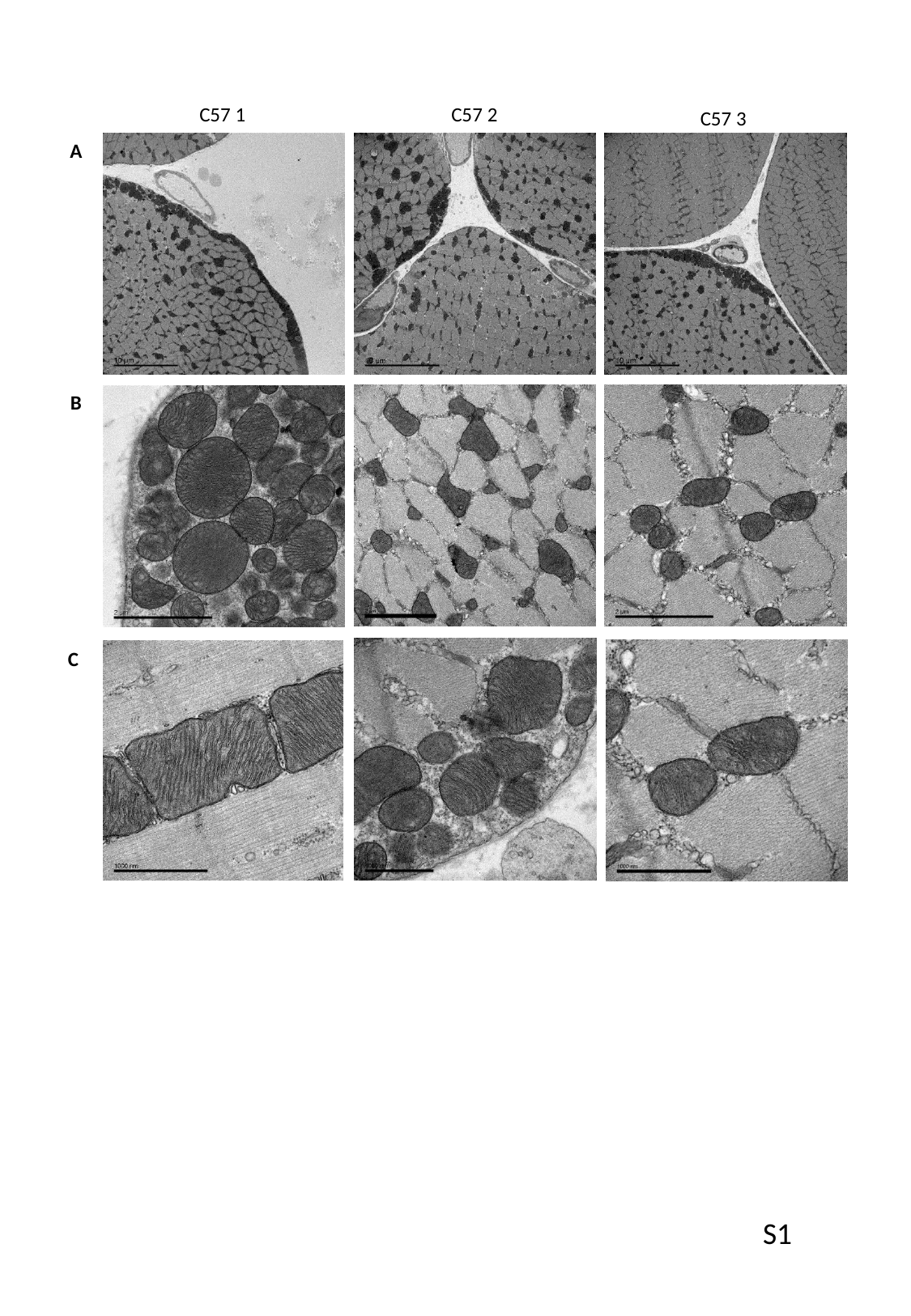

C57 1
C57 2
C57 3
A
B
C
S1

Supplement: Supplementary file 1 — Transmission electron micrographs of tibialis anterior (TA) muscles from C57BL/10 (C57) mice. Cross-sections of TA muscles from C57 mice. Scale bars = (A) 10 μm, (B) 2 μm and (C) 1000 nm. (n = 3; C57 1, C57 2, C57 3). (PPTX 787 kb) [file 13395_2017_139_MOESM1_ESM.pptx]

## Slide 1
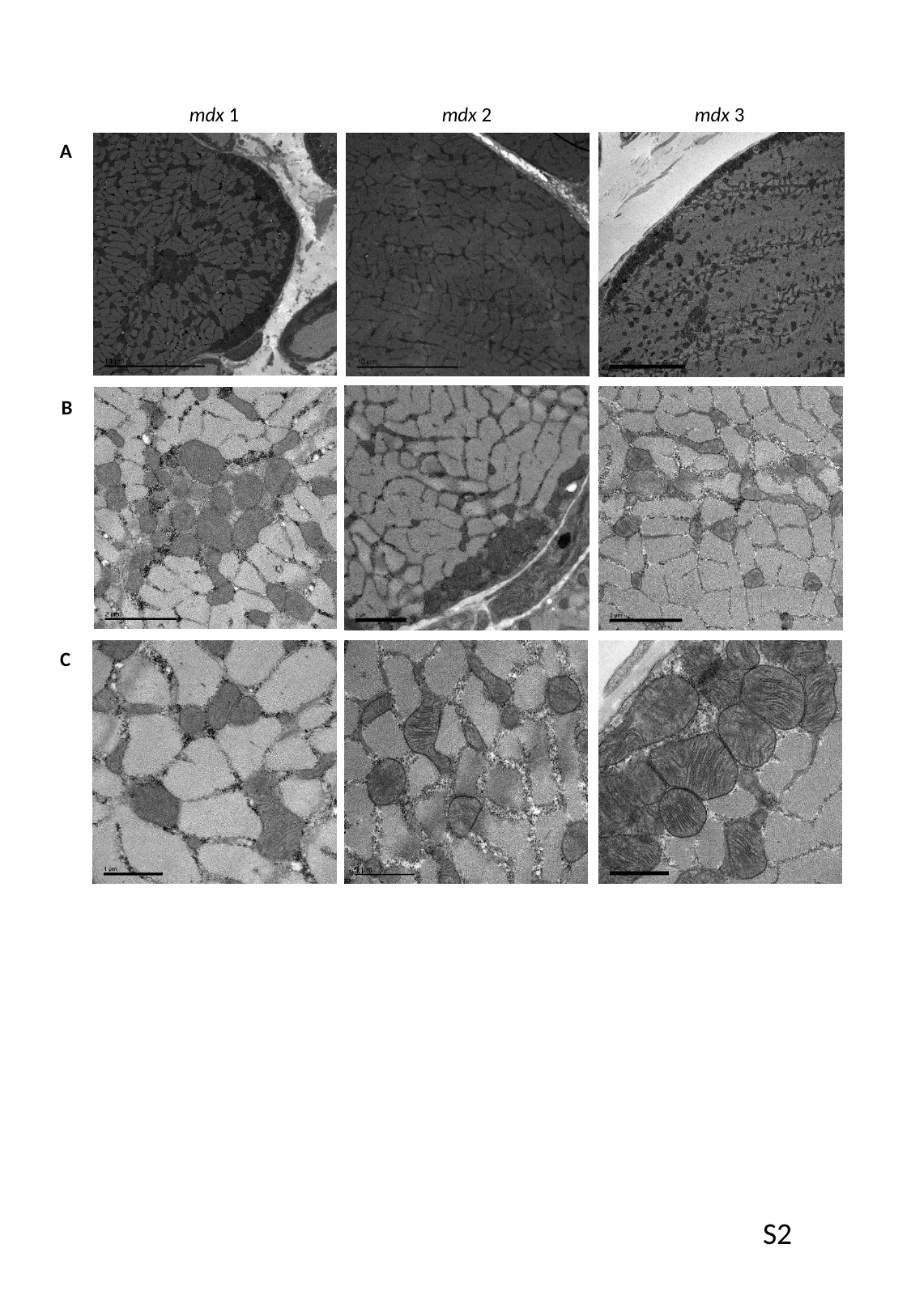

mdx 1
mdx 2
mdx 3
A
B
C
S2

Supplement: Supplementary file 2 — Transmission electron micrographs of tibialis anterior (TA) muscles from mdx mice. Cross-sections of TA muscles from mdx mice. Scale bars = (A) 10 μm, (B) 2 μm and (C) 1000 nm. (n = 3; mdx 1, mdx 2, mdx 3). (PPTX 805 kb) [file 13395_2017_139_MOESM2_ESM.pptx]

## Slide 1
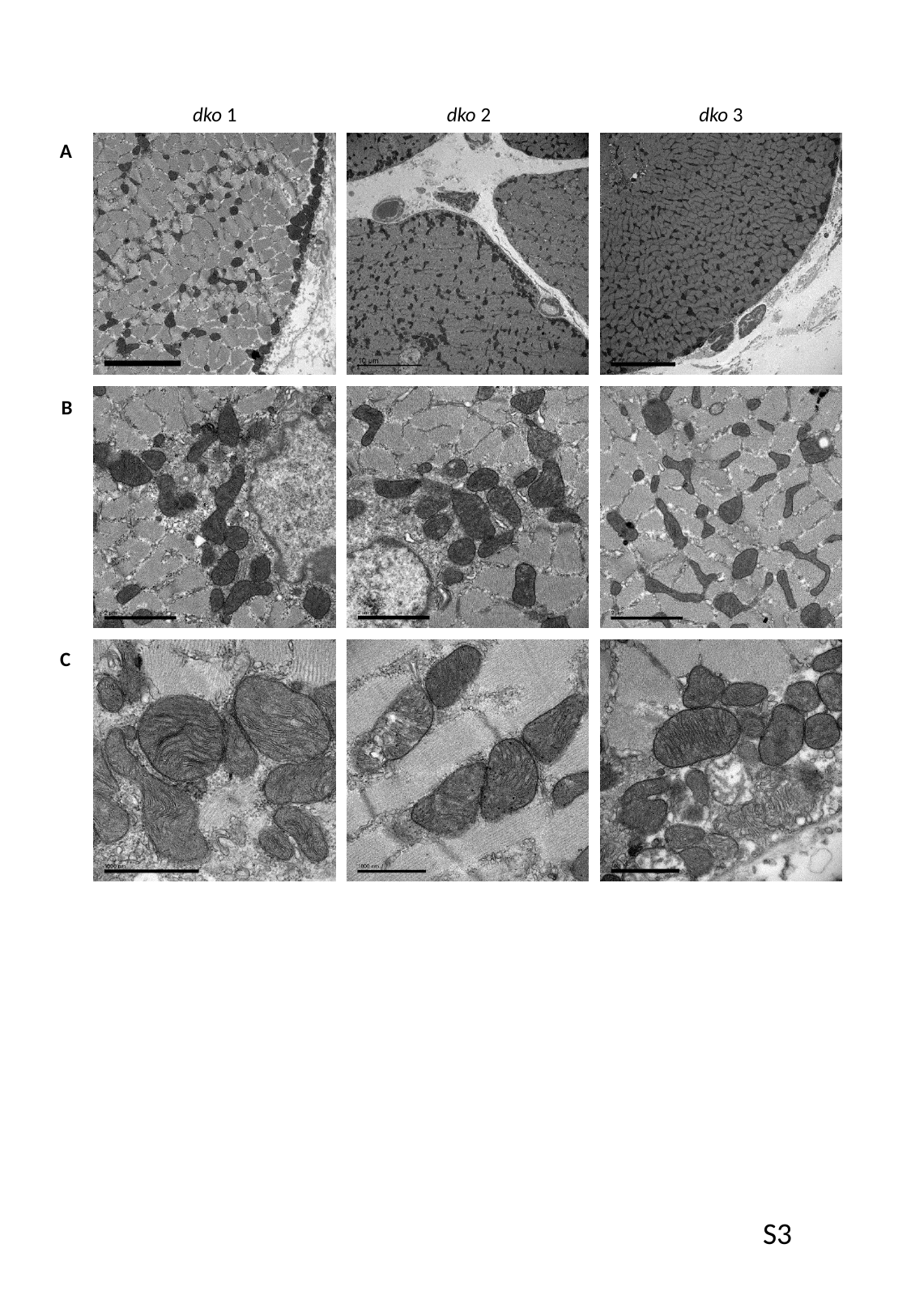

dko 1
dko 3
dko 2
A
B
C
S3

Supplement: Supplementary file 3 — Transmission electron micrographs of tibialis anterior (TA) muscles from dko mice. Cross-sections of TA muscles from dko mice. Scale bars = (A) 10 μm, (B) 2 μm and (C) 1000 nm. (n = 3; dko 1, dko 2, dko 3). (PPTX 841 kb) [file 13395_2017_139_MOESM3_ESM.pptx]

## Slide 1
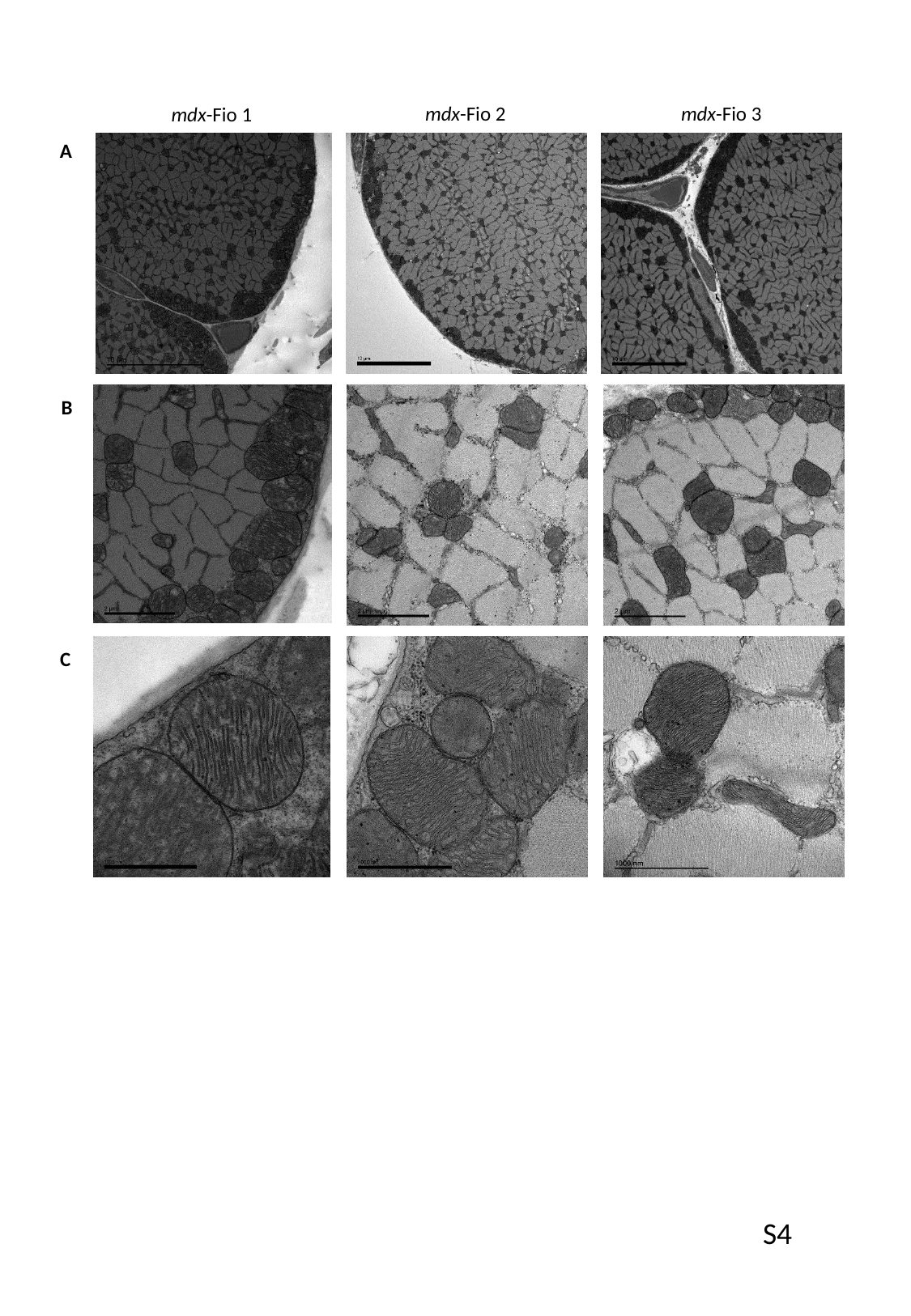

mdx-Fio 2
mdx-Fio 3
mdx-Fio 1
A
B
C
S4

Supplement: Supplementary file 4 — Transmission electron micrographs of tibialis anterior (TA) muscles from mdx-Fiona (mdx-Fio) mice. Cross-sections of TA muscles from mdx-Fio mice. Scale bars = (A) 10 μm, (B) 2 μm and (C) 1000 nm. (n = 3; Fio 1, Fio 2, Fio 3). (PPTX 771 kb) [file 13395_2017_139_MOESM4_ESM.pptx]
